# Automated Analysis of NF-κB Nuclear Translocation Kinetics in High-Throughput Screening

**DOI:** 10.1371/journal.pone.0052337

**Published:** 2012-12-27

**Authors:** Zi Di, Bram Herpers, Lisa Fredriksson, Kuan Yan, Bob van de Water, Fons J. Verbeek, John H. N. Meerman

**Affiliations:** 1 Division of Toxicology, Leiden/Amsterdam Center for Drug Research, Leiden University, Leiden, The Netherlands; 2 Imaging & BioInformatics, Leiden Institute of Advanced Computer Science, Leiden University, The Netherlands; Rutgers University, United States of America

## Abstract

Nuclear entry and exit of the NF-κB family of dimeric transcription factors plays an essential role in regulating cellular responses to inflammatory stress. The dynamics of this nuclear translocation can vary significantly within a cell population and may dramatically change e.g. upon drug exposure. Furthermore, there is significant heterogeneity in individual cell response upon stress signaling. In order to systematically determine factors that define NF-κB translocation dynamics, high-throughput screens that enable the analysis of dynamic NF-κB responses in individual cells in real time are essential. Thus far, only NF-κB downstream signaling responses of whole cell populations at the transcriptional level are in high-throughput mode. In this study, we developed a fully automated image analysis method to determine the time-course of NF-κB translocation in individual cells, suitable for high-throughput screenings in the context of compound screening and functional genomics. Two novel segmentation methods were used for defining the individual nuclear and cytoplasmic regions: watershed masked clustering (WMC) and best-fit ellipse of Voronoi cell (BEVC). The dynamic NFκB oscillatory response at the single cell and population level was coupled to automated extraction of 26 analogue translocation parameters including number of peaks, time to reach each peak, and amplitude of each peak. Our automated image analysis method was validated through a series of statistical tests demonstrating computational efficient and accurate NF-κB translocation dynamics quantification of our algorithm. Both pharmacological inhibition of NF-κB and short interfering RNAs targeting the inhibitor of NFκB, IκBα, demonstrated the ability of our method to identify compounds and genetic players that interfere with the nuclear transition of NF-κB.

## Introduction

**Figure 1:**
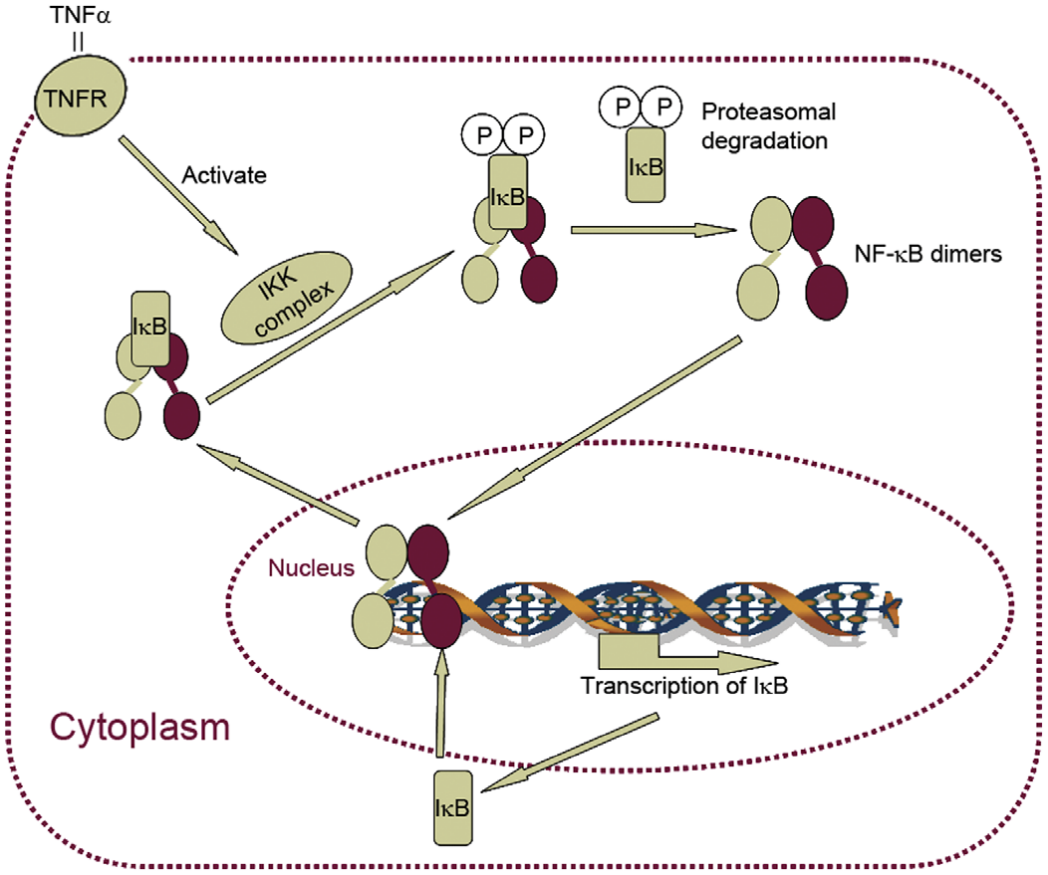
NF-κB oscillation is regulated by an auto-regulatory negative feedback loop. Simplified schematic overview of the TNFα-induced canonical NF-κB response. TNFα binding to the TNF receptor (TNFR) activates the inhibitor of kappa-B kinase (IKK) complex, leading to phosphorylation of the inhibitor of NF-κB, IκB, upon which NF-κB is free to enter the nucleus to activate transcription of its target genes. One of the primary NF-κB target genes is IκB, which may retrieve NF-κB from the nucleus to maintain inactive IκB::NF-κB complex in the cytoplasm. Ongoing TNFR signaling can re-initiate the induction-inhibition cycle.

NF-κB is a family of dimeric transcription factors consisting of homo- or heterodimers of different subunits (e.g. p65/RelA). It is involved in cellular stress responses to stimuli such as cytokines, free radicals, ultraviolet irradiation, oxidized LDL, and bacterial or viral antigens [1], [2], [3], [4], [5]. In resting cells, NF-κB dimers are located within the cytoplasm, bound to the NF-κB inhibitor IκB. After NF-κB–activating stimuli such as TNFα or IL1β, the IKK (the inhibitor kappa B kinase) complex is activated, which in turn phosphorylates IκB [6] and NF-κB [7], [8]. Phosphorylated IκB proteins are then ubiquitinated and degraded by the proteasome, thereby liberating NF-κB dimers that translocate into the nucleus and regulate the transcription of the target genes. However, NF-κB dimers do not stay in the nucleus permanently. IκBα, a member of IκB family, is a transcriptional target of NF-κB [9]. Therefore, transcription of IκBα creates a negative feedback loop: newly synthesized IκBα protein enters the nucleus and binds to NF-κB, leading to the export of complex back to the cytoplasm (Figure 1). This negative feedback loop creates an oscillation of NF-κB nuclear-to-cytoplasmic translocation. Such a response seems essential in modulating differential transcriptional responses under transient or sustained cytokine signaling [10]. Given the role of NF-κB in diverse (patho)physiological responses, understanding the cell population dynamics of this process is essential.

**Figure 2:**
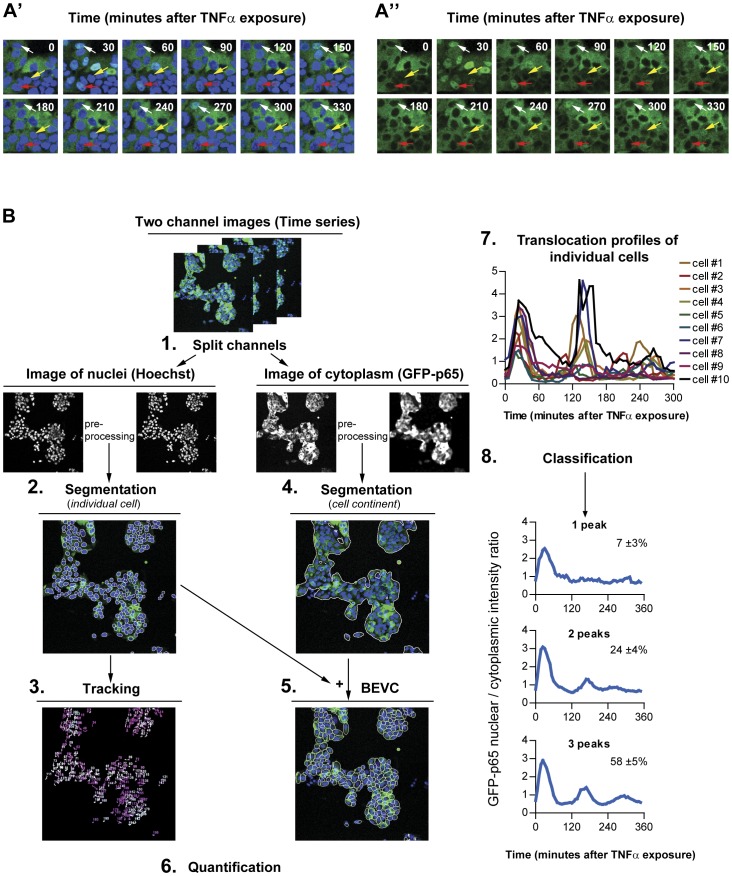
Image-based NF-κB nuclear translocation analysis. Time series images of GFP-p65 expressing HepG2 cells stimulated with 10 ng/mL TNFα. (A') Nuclear channel. (A'') GFP-p65 channel. Examples of multiple nuclear translocations at 30, 150 and 270 minutes (white arrow) and at 30, 120, 210 and 330 minutes (yellow arrow). Example of only one, long, nuclear translocation event (red arrow). (B) Flowchart of the individual cell NF-κB nuclear translocation analysis. 1. Splitting of the two-channel image time series of the NF-κB response 2. Nuclear image preprocessing and segmentation. 3. Tracking of nuclear mask throughout the time series. 4. Segmentation of cell locations. 5. Definition of the best ellipse fitting within a Voronoi cell (BEVC) as the cytoplasmic mask. 6. Quantification of the ratio of the nuclear and cytoplasmic GFP intensity per time-point, per cell. 7. Analysis of the nuclear translocation profile of individual cells. 8. Categorization of responses to perform population analyses.

The most common approach taken in NF-κB translocation studies, which simply measures the NF-κB localization ratio between the total nuclear and the total cytoplasmic region, obscures the fact that not all cells respond to the stimulation synchronously [10], [11], (Figure 2A’ and 2A”). Similarly, recent studies of lipopolysaccharide-induced NF-κB activity showed that only half of the cells responded to the secondary TNFα autocrine signal, creating distinct subpopulations [12], [13]. Such cell-to-cell heterogeneity seems essential for the plasticity of tissue responses to inflammation [14], [15].

Furthermore, NF-κB responds to many different stimuli, each of which may lead to different activation dynamics. To understand NF-κB signaling under a wide variety of stimulation conditions, it is important to measure single-cell NF-κB dynamics in large cell populations. Obviously, studies of NF-κB translocation in just several individual cells are not sufficient for this purpose, although dedicated and sophisticated image analysis methods have been developed for this specific task [11], [16], [17]. In order to systematically determine factors that define NF-κB translocation dynamics, high-throughput screens need to be developed in relevant cell lines in the context of compound screening and functional genomics.

Our goal was to develop a methodology for quantification of NF-κB translocation dynamics in single cells, suitable for high throughput screening (HTS). For this, we used HepG2/GFP-p65 cells which show a dynamic nuclear-to-cytoplasmic translocation response upon TNFα stimulation (Figure 2A). To derive quantitative information of this shuttling in the entire cell population, we set out a strategy for the image analysis (Figure 2B). We describe two novel segmentation methods that are required for this purpose: one for the segmentation of individual nuclei, and one for the cell region. Next, cell tracking was done based on the nuclear segmentation results. Finally, methods for the quantification of NF-κB translocation dynamics and the extraction of informative parameters from the NF-κB translocation time profiles are described. In addition, procedures and results for the validation of each step in the quantification methodology are presented.

## Results

### Image Collection and Preprocessing

**Figure 3:**
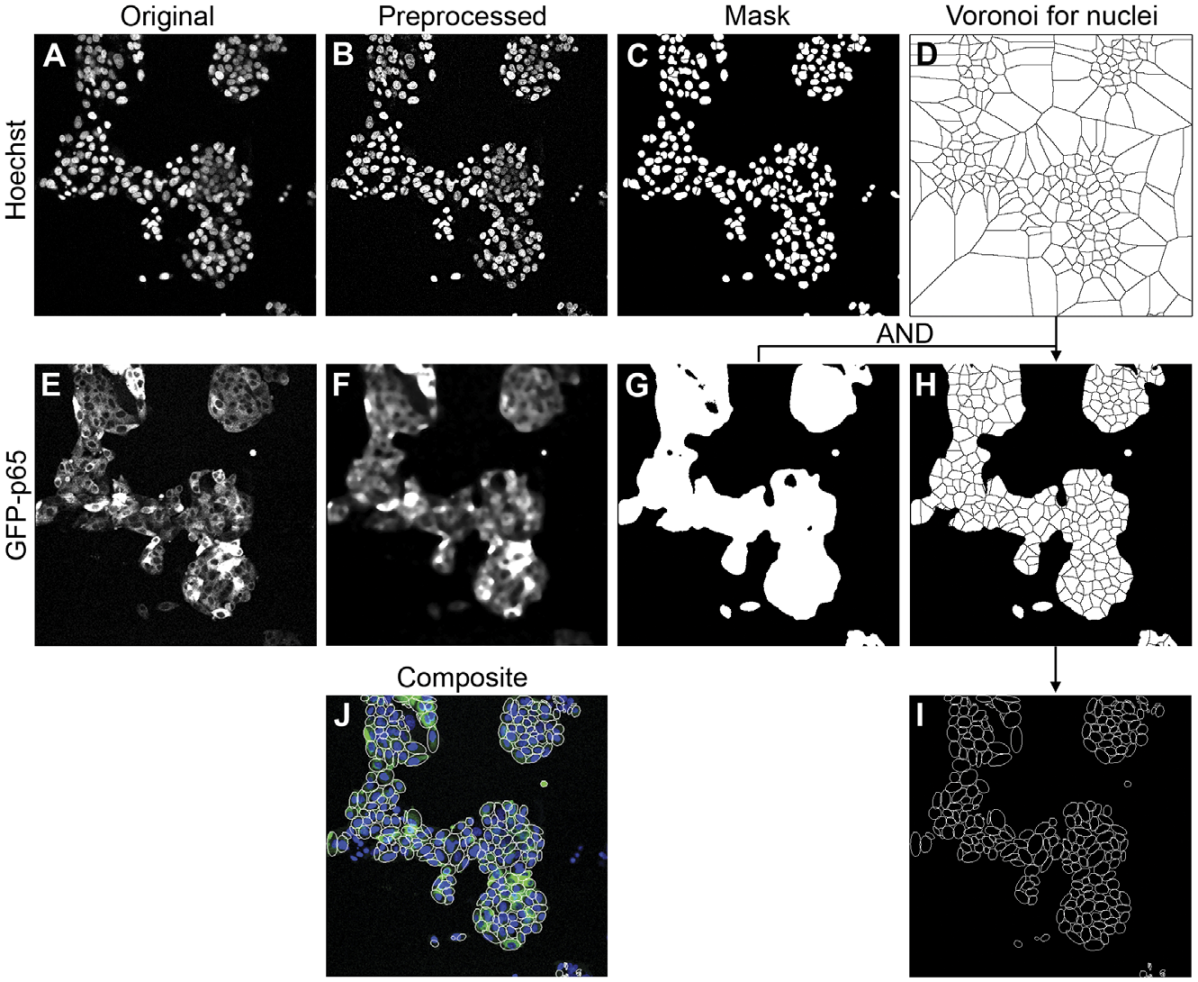
Stepwise demonstration of the image analysis method. The original nuclear Hoechst channel (A) is pre-processed by image sharpening and background subtraction (B), followed by WMC and nuclear mask definition (C). Subsequently, the Voronoi diagram (D) is generated based on the disjointed nuclear masks. For the GFP-p65 channel, the original image (E) is preprocessed by a smoothing filter (F) for global cell location definition (G). By multiplication of the global cell masks (G) with the Voronoi diagram (D), the Voronoi mask is defined for the each cell (H). Within each Voronoi masks the cytoplasmic areas are redefined as the best-fit ellipse in each Voronoi cell (I). Figure (J) shows the composite view of original Hoechst channel, GFP-p65 channel and the BEVC segmentation result.

First, dual channel confocal images were collected (first channel: Hoechst nuclear staining; second channel GFP-p65) in a six hour time-lapse series with a recording interval of 6 minutes (see Materials and Methods for details). Next, image preprocessing was applied separately for each of the two channels (Figure 3A and 3E respectively). For the nuclear channel, images were sharpened first in order to enhance the edge (by ImageJ; http://rsbweb.nih.gov/ij/). This was implemented by an unsharp filter which equals to subtracting a Gaussian blurred copy of the image and rescales the image to obtain the same contrast of large (low-frequency) structures as in the input image. We empirically defined the optimal radius of the Gaussian filter [18] to be 3.0, and the scaling of the filter 0.6. Next, the so-called Rolling Ball method [19] was used to remove unevenly illuminated background by subtracting an averaged image intensity within a circular kernel around each pixel (by ImageJ). The size of the kernel was chosen to be slightly larger than the radius of the largest nucleus. The pre-processed image of the nuclear channel is shown in figure 3B. To define the overall cell region in the images, the GFP-p65 channel was processed with a Median filter [20], resulting in smooth cellular regions (Figure 3F).

### Nuclei Segmentation: Watershed Masked Clustering (WMC)

The segmentation of the nuclei was accomplished by watershed masked clustering [21], [22]. This method uses a watershed segmentation to divide images into separated regions containing one nucleus per region. Subsequently, within each region K-means clustering [23] was applied to define the nuclear region (Figure 3C). This method is based on the assumption that each nucleus is evenly illuminated and the contrast between nuclei and background is sufficiently high. Over-segmentation is a well-known issue of watershed segmentation. In order to address this issue, preprocessed images (Figure 3B) were convolved with a Gaussian filter to smooth discrete intensity signals, using an optimized kernel size. Once watersheds were obtained from this image, the preprocessed images prior to Gaussian convolving (Figure 3B) were used to apply K-means clustering.

### Cell Tracking

The nuclear masks that were obtained from the segmentation were used for the cell-tracking. In our NF-κB translocation experiments, we observed that most of the cells moved over short distances between two consecutive image frames, and also a negligible number of cell divisions occurred during the image acquisition period. Given these conditions, the maximum overlap ratio (OLR; See Equation 1) is a feasible and applicable criterion. For every labeled nuclear region in the current frame 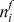, where *i* represents corresponding label and *f* represents the frame index, we identified the labeled nucleus in the next frame 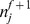 which maximizes with 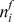:

**Equation 1:** Overlap Ratio.
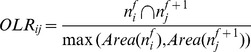



Given the short imaging interval, cells should not disappear from one frame to another except when moving out of the frame borders. Disappearing cells are thus likely to be caused by under-segmentation. In order to avoid fragmented cell traces, as may occur in the more condensed cell clusters, we applied an extra tracking image buffer [24] to store disappearing cell regions until they are recovered again in one of the subsequent frames that maximizes the OLRs. As a result, nuclei that are not consistently detected in every frame can still be tracked correctly.

### Cell Segmentation: Best-fit Ellipse of Voronoi Cell (BEVC)

The objects that need to be extracted in this particular live cell NF-κB imaging application are cells that grow in clusters. These cells touch and may slightly overlap with each other thus making it sometimes difficult to uniquely identify the cellular edges. Therefore the classical edge detection methods which locate the maximum intensity gradient are not applicable to this particular case. Instead, we propose a single cell simulation algorithm called best-fit ellipse of Voronoi cell (BEVC). The algorithm produces an estimate of the single cellular areas based on the topology of the cells, which is derived from the distribution of nuclei. In principle, it consists of three steps:

**Step 1, general topology of cell culture.** A Voronoi diagram [25] was generalized based on a set of disjointed nuclear masks 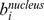 for *i* = 1,2,3,…,D, (Figure 3C) with 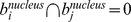 when 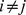, where D is number of nuclear masks. Each Voronoi cell *V*_i_ containing 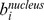 (Figure 3D) is defined as a region which includes all pixels *r* closer to the boundary of 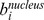 than to the other nuclear masks. The formula is presented as following:

**Equation 2:** Voronoi Cell.
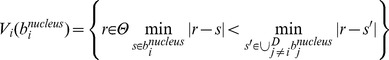



**Step 2, obtain the Voronoi diagram for the cluster of cells.** A global threshold was applied to the preprocessed images of the GFP-p65 channel (Figure 3F) to obtain the binary masks (Figure 3G). Subsequently the masks were multiplied (AND) with the Voronoi diagram from step 1, so that only the Voronoi cells within the binary mask were preserved (Figure 3H).

**Step 3, obtain an estimate of cell shape per Voronoi cell.** The underlying model for BEVC is that cells are ellipsoid shaped objects. Based on this assumption, we simulated the region, or better, shape, of an individual cell as the best-fit ellipse in each Voronoi cell *V*_i_ by calculating the major and minor axis from the centralized moments (Figure 3I) [26], [27].

### Quantification of NF-κB Translocation Dynamics

Prior to establishing NF-κB translocation dynamics profile, both cellular masks and nuclear masks were validated by a supervised two-class classifier, based on morphological features (Table S1), in order to exclude improper segmentation (file S1, Figure S2, Figure S3). The training dataset consisted of manually discerned masks (file S1, Figure S1). For each single cell *i*, the NF-κB translocation dynamics is defined as a time-profile of the ratio of average intensities of nuclear area 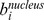 and cytoplasmic area, the latter defined as total cellular area minus nuclear area 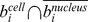


**Equation 3**: NF-κB translocation dynamics of single cell *i* on time point *t.*

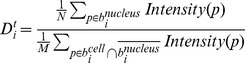
 where 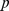 represents pixel. N is the number of pixels in the nuclear mask of cell *i*, and M is the number of pixels in the cytoplasmic mask of cell *i*.

For cells with tracks that disappeared in 3 or less than 3 consecutives frames, linear interpolation was applied to generate missing data. For cells with tracks that disappeared in more than 3 consecutive frames, interpolation becomes too inaccurate and the corresponding translocation profiles were removed from the final data. Generally, <30% of cells were removed by this procedure.

### Quantification of NF-κB Translocation Analogue Parameters

One advantage of the proposed analysis method is its ability to automatically quantify analogue parameters for each individual translocation profile (File S1, Figure S5, Table S2).

We first defined all translocation events. These start at a local minimum of a profile, include the next local maximum, and end at the next local minimum. We calculated the number of translocation events, various properties for each translocation event, nuclear entry and exit rates and time between consecutive peaks; in total 26 analogue parameters. More detailed information and pseudo code are presented in the File S1,

### Statistical Validation of the NF-κB Quantification Method

We validated our quantification method in a 3 step process using 5 randomly selected time lapse movies. First, we compared our BEVC method for cell segmentation with other segmentation methods that are used to segment touching or overlapping cells. One approach that is often used to define the cytoplasmic topological region is to dilate the corresponding nuclear segmentation mask by a few iterations. However, the extent of the dilation requires fine-tuning for different cell sizes to avoid overlap between individual cells. Another approach is to define the cell region by only applying the Voronoi diagram. Our method (BEVC) extends the topology information from the Voronoi diagrams with a best-fitting ellipse, which leads to a more stringent definition of the cellular area.

To compare these three methods (Dilation, Voronoi and BEVC), we first generated the binary images from the different methods. For the dilation, we used a circular kernel with a radius of 3 pixels as a cytoplasmic structure element, based on the general cell size in our images. Next, we assessed each result by human perception. For this, 5 test frames from different series were used with a total of 1116 nuclei. For each frame *f*, a score named “error rate” was calculated to measure the segmentation accuracy:

### Equation 4: Error Rate


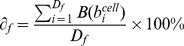
 for binary indicator 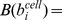
_{
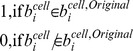
_where 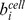 is the cellular mask of cell *i* obtained from one of three methods. 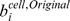 is the cellular mask of *i*'th cell obtained by human perception. 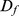 is the total number of cells in image frame *f*, calculated by one of three methods.

**Figure 4:**
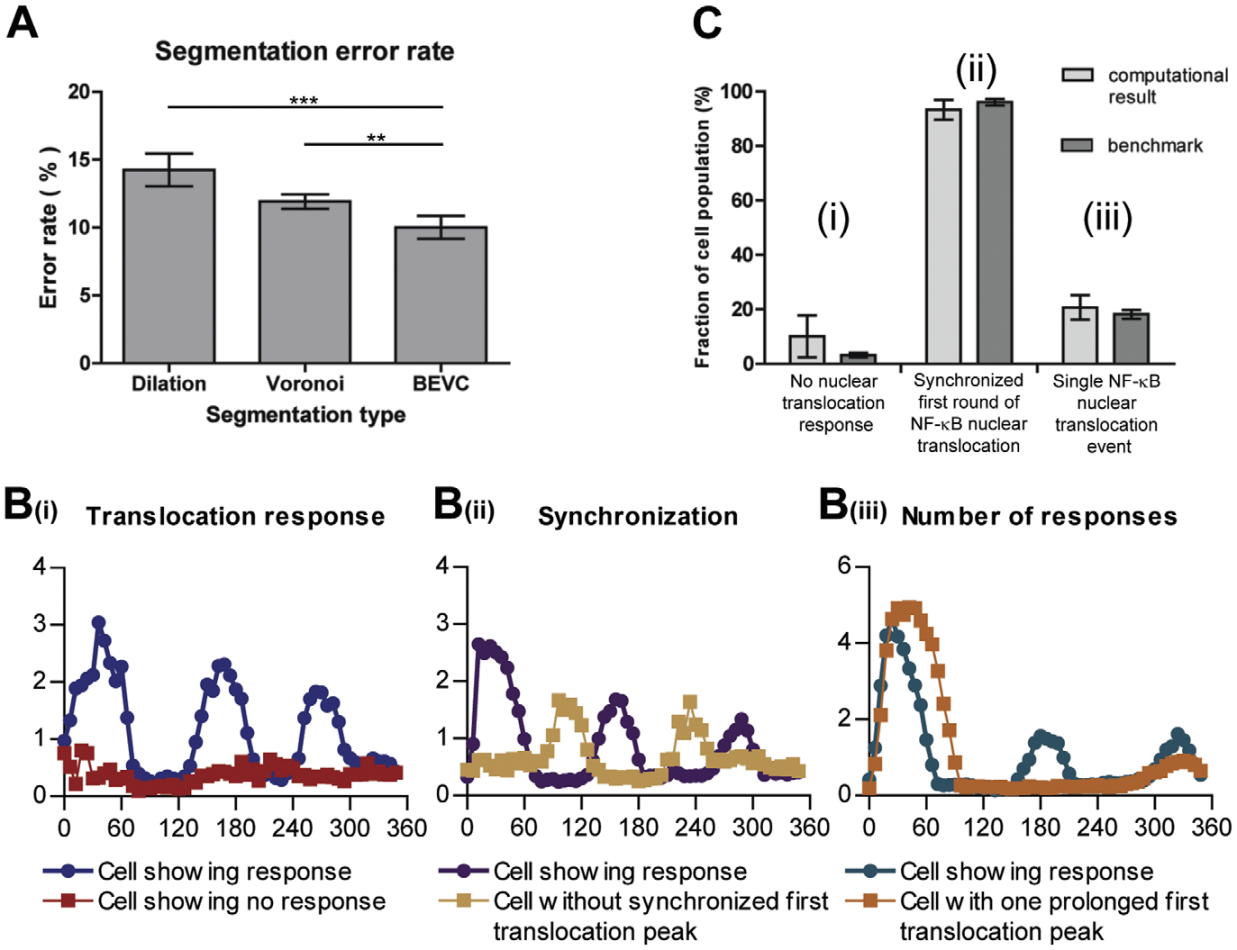
Statistical validation of the automated image segmentation and NF-κB translocation quantification. (A) Comparison of 3 cytoplasmic segmentation methods based on the criterion of error rate. The error rate of the Dilation method is 14.5%±3.2; of Voronoi it is 11.8%±1.4; and of BEVC it is 10.3%±2.2.* p-value<0.05; ** p-value <0.005; Paired t test (B) Example translocation profiles of *(i)* cells without translocation and cells with translocation, *(ii)* cells with and without a synchronized first round of NF-κB translocation, *(iii)* cells with NF-κB translocation occurring only once and cells with more than one NF-κB translocation event. (C) Bias assessment of our quantification method by comparison of the computational results with the benchmark for different subpopulation. No significant differences (P-value >0.1) were found between the computational results and the benchmark for the different cell subpopulations within a 6 hour imaging timeframe.

The use of the Voronoi combined with best fit ellipse (BEVC) yielded the smallest error rate for cytoplasmic area definition (10.3%±2.2%), compared to a dilation or Voronoi method (14.5%±3.2% and 11.8%±1.4% respectively) (Figure 4A).

Next, we validated our NF-κB translocation quantification method by comparing automatically generated translocation profiles with a benchmark that was produced from cells with segmentation and tracking profiles that were validated by human perception. 5 randomly selected time lapse movies each with 47 frames were used in this test. From each test movies, 3 benchmarks were generated separately by 3 independent individuals (File S1, Figure S4), in order to compensate for possible human bias. Subsequently, a split-plot ANOVA [28] was applied (by Statistical Computing Seminars Repeated Measures Analysis with R) to test for the difference between the benchmark profiles generated by the 3 test persons and the computational result, in total 4 groups. The metric is the NF-κB Nuclear/Cytoplasmic intensity ratio, and 2 independent factors are time and group. The statistical tests indicate that the variation between the 3 benchmarks is not significant; moreover there are no significant differences between the benchmarks and the computational result (File S1, Figure S4). This indicates that the designed algorithm provides an accurate estimation of NF-κB translocation profiles.

### Computational Efficiency of the Algorithm

We tested computational efficiency of the algorithm on the dataset obtained from HepG2/GFP-p65 cells (see Materials and Methods for details). The computational complexity of this algorithm is O(nlogn). We analyzed 6 sets of 60 time-lapse movies (6 times 3.51 GB). Each 512×512 movie contains two channels, and each channel consists of 60 frames. On average, 250 cells were analyzed per movie. The analysis of this dataset was completed in 83±2 minutes on a desktop PC (Intel Core i7-3770, 3.40 GHz with 8 GB of RAM and Microsoft Windows 7 Professional, SP1). The computationally most intensive part is the background subtraction on the nuclear channel followed by the segmentation of the nuclei by WMC. This takes ∼64 seconds per movie. Tracking of the nuclei is done in 6 to 7 seconds.

### Statistical Validation of Cell Population NF-κB Dynamics

One of the main purposes of quantifying single-cell NF-κB nuclear translocation dynamics, especially in the context of high-throughput screens, is to study the heterogeneity between cell subpopulations. Therefore it is necessary to validate whether our quantification method correctly identifies specific sub-populations of cells and does not create a bias towards any particular cell population. To establish this, we benchmarked 5 time series images (with 1116 cells) by manually counting the cell subpopulations. We performed three separate tests, comparing the computational results with the benchmark. In each test, cells were clustered into two complementary categories. In the *first* test, cells were clustered in cells without translocation response versus cells with translocation response (Figure 4B(i)). In the *second* test, we distinguished cells with a synchronized first peak of NF-κB translocation, from non-synchronized responders (Figure 4B(ii)). In this category, synchronization was defined as the first NF-κB translocation peak occurring within three frames from the averaged profile. The *third* test clustered cells into (a) cells with only one (prolonged) NF-κB translocation event, and (b) cells with more than one NF-κB translocation event (Figure 4B(iii)). The reason for defining these three tests is their simplicity for human counting. For all three tests, we obtained p-values greater than 0.1, indicating that there is no significant difference between our computational result and the benchmark. Therefore, we conclude that our algorithm can efficiently be used to perform population studies on NF-κB nuclear translocation profiles.

### Biological Validation of the NF-κB Quantification Method

**Figure 5:**
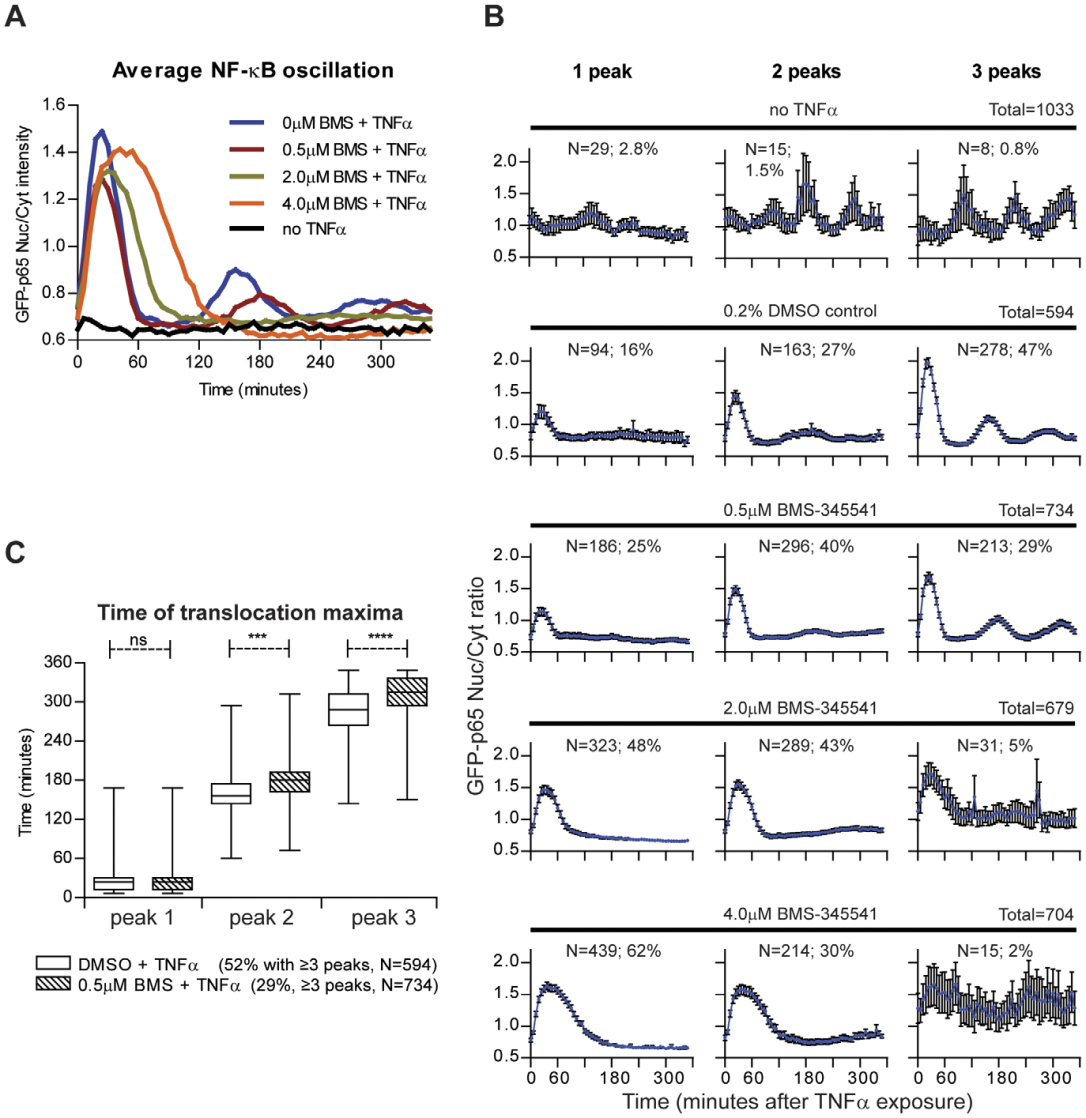
Population analysis of NF-κB nuclear translocation perturbation by the IKKβ inhibitor BMS-345541. Cells were pre-treated for 2 hours with increasing concentrations of BMS-345541 before TNFα stimulation. (A) Average nuclear translocation response graphs, calculated from the translocation profiles of individual cells. (B) Average nuclear translocation response graphs with standard error bars for cells with one, two or three translocation peaks. The total number of cells, the number (n) and percentage of cells which show responding number of peaks are presented (C) Analysis of the time distribution of the median of 1^st^, 2^nd^ and 3^rd^ nuclear translocation maximum in TNFα stimulated and TNFα stimulated plus 0.5 µM BMS pre-treated cells. ns: No significant difference; *** p-value <0.001; **** p-value <0.0001.

In order to establish the sensitivity of our algorithm for perturbation of the biological system, a pilot experiment was performed by pre-exposing the HepG2/GFP-p65 cells for 2 hours with increasing concentrations of an IKK-inhibitor, BMS-345541 (0.5, 2.0 and 4.0 µM) before TNFα stimulation. Inhibition of IKK will prevent NF-κB nuclear translocation (see Figure 1). The experiment was performed in 96-well plates on two different days, with two replicates per plate. In the first analysis step, the average GFP-p65 nuclear/cytoplasmic ratio profiles were generated from our quantification method. Already at very low inhibitor concentrations (0.5 µM), the second and third NF-κB nuclear translocation maxima were delayed and the amplitude of the first peak was decreased. Increasing the concentration of BMS-345541 to 2.0 and 4.0 µM prolonged the first nuclear translocation event. Without TNFα stimulation, no NF-κB oscillation was observed (Figure 5A).

Next, the individual GFP-p65 nuclear translocation profiles were analyzed for the number of translocation events within the 6 hours imaging period after TNFα stimulation. In non-stimulated cells, 5% of the cells show spontaneous nuclear translocation, which is non-synchronous (Figure 5B). After TNFα stimulation, there is nuclear translocation with either one, two or three peaks, in 90% of the cells (Figure 5B). The average nuclear translocation response graphs for individual cells with either one, two or three peaks, clearly show that the percentage of cells with only one transition peak increases with the concentration of BMS-345541 (Figure 5B), and that the percentage of cells with 3 transition peaks decreases.

In addition, we compared the time distribution of each translocation maximum between control and BMS-345541 pre-treatment. This indicated that already at a low concentration (0.5 µM) a significant delay occurred for the second and third translocation maxima (Figure 5C).

In conclusion, these data indicate that the quantification method can be used to perform cell-population studies, to identify rare events, and to study drug-dependent effects, even at low concentrations.

### Application of the NF-κB Quantification Method in High Throughput Screening Assays

Having validated our NF-κB nuclear translocation quantification approach for segmentation accuracy, for correct sub-population analysis, and for sensitivity to biological perturbation of the system, we validated whether our quantification method can successfully be applied in the context of high-throughput functional genomics screening. For this screening, the approach of gene silencing by transient transfection of short interfering RNAs (siRNAs) was applied. We used three different siRNAs as control: positive control siNFKBIA that targets IκBα, upon which knockdown the NF-κB response will be affected [11]; negative control siCASP8 that targets caspase 8, which is a downstream effector of the TNFR, but does not affect the NF-κB activation; and siRNA control #1 (targeting luciferase) which also should not affect the NF-κB activation These siRNAs were tested in 12 different 96-well plates (2 replicates per plate) on 4 different days, allowing an accurate analysis of the robustness of the assay.

**Figure 6:**
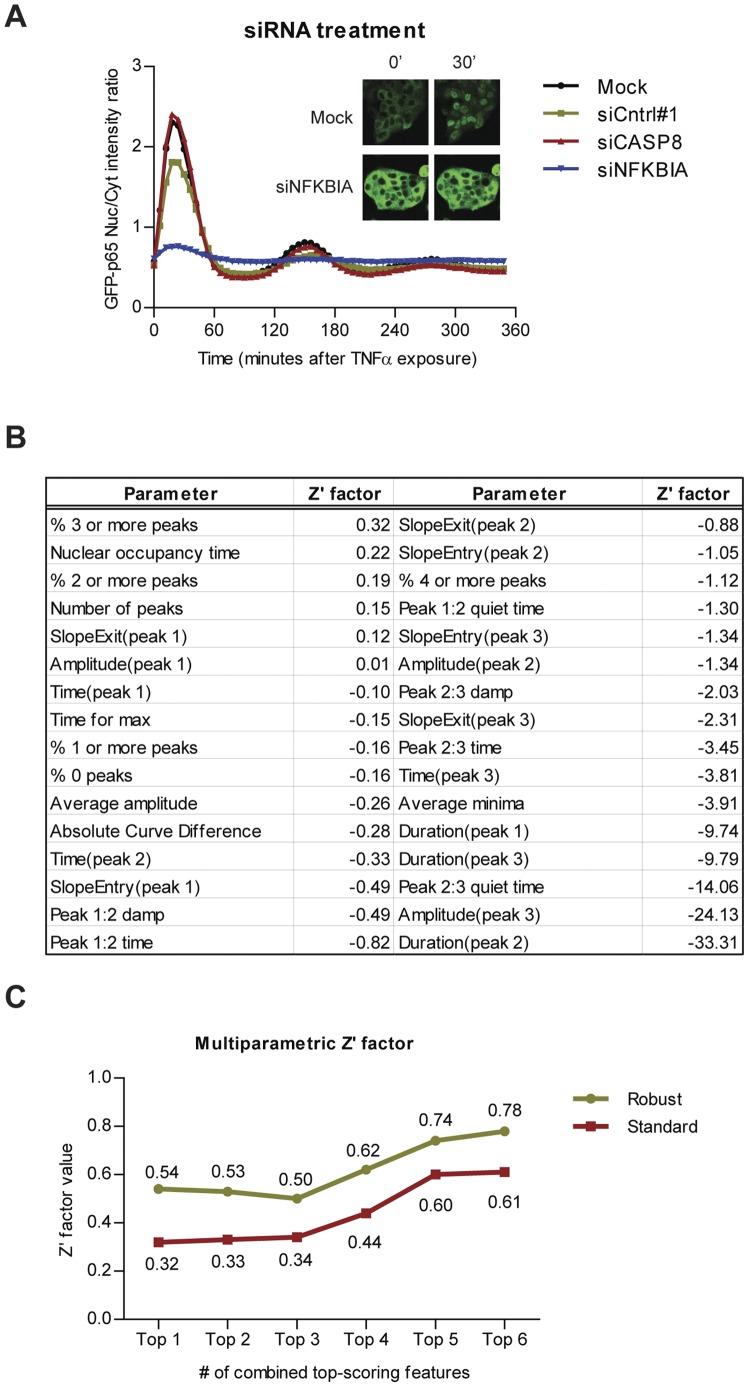
Application of the individual cell NF-κB nuclear translocation analysis in siRNA screening assays. (A) The average nuclear translocation response graphs for negative controls siCASP8, siCntrl#1, transfection reagent without siRNA (mock), and positive control siNFKBIA. Inset: representative images of mock and siNFKBIA treated GFP-p65 cells, at 0 and 30 minutes after TNFα stimulation (B) Table showing the univariate Z'-factors of all 32 individual parameters. The definitions of the 26 analogue parameters are given in **Table S2**. Absolute Curve Difference: the absolute point-by-point difference between control and treatment averages. (C) Multivariate Z'-factor calculation based on top-scoring univariate Z'-factors. Both the conventional as well as the robust multivariate Z'-factors exceed the confidence threshold of 0.5 by combining ≥5 top-scoring univariate Z'-factors by linear projection.

First, we calculated the average GFP-p65 nuclear/cytoplasmic ratio profiles for each control, as well as for the cells that were not transfected with siRNAs, but exposed to the transfection reagent (mock) (Figure 6A). We did not detect an effect of caspase 8 knockdown on NF-κB oscillation compared to mock treatment; yet surprisingly, siCntrl#1 slightly decreased the peak amplitude. IκBα knockdown however, strongly impaired NF-κB oscillation as expected.

Next, for each well, we calculated the average of the 26 analogue parameters for the cell population and derived further sub-population information, such as the average GFP signal intensity, the absolute difference between treatment and control graphs, as well as the percentage of cell profiles showing 0 to 4 transitions: in total 32 parameters (Figure 6B). To validate the reproducibility of the controls and the quality of assay, we calculated the Z'-factors which quantify the stability of both positive and negative control, as well as the distance between positive and negative controls [29] for each individual parameter. The conventional methods for assay quality control and hit identification in high-throughput functional genomics screens were developed for assays with only a single readout; however, our quantification method provides readouts for multiple parameters. To enable the comparison of our assay with assays using only a single readout, we integrated multiple parameters into one value by Fisher’s linear discriminant [30], [31] as suggested recently for integration of multiple readouts for quality control in high-content screening [32]. Before calculating the Z'-factors, all the data were normalized to the plate average and plate standard deviation by calculating the Z-score (the number of standard deviations from the mean). In order to calculate Z'-factors, a direction 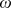 was first identified where maximum separation between positive and negative control occurs (Equation 5). The multidimensional data were then linearly projected onto this dimension (Equation 6) and a multivariate Z'-factor can be calculated from the projected values. We calculated both classical Z'-factors and robust Z’-factors (Equation 7) by estimating the mean and standard deviation, and the median and median absolute deviation (MAD), respectively.

**Equation 5**: Projection direction.
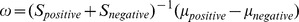
Where 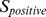 and 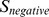 is the covariance matrix of positive control and negative control. 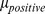 and 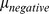 is the mean vector of positive control and negative control.

**Equation 6**: Linear projection of multi-parametric dataset.
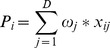
Where 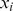 is a multi-parametric vector with D parameters.

**Equation 7**: Robust Z'-factor.
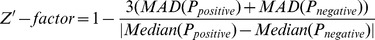



The univariate Z'-factors for all 32 parameters are presented in the Figure 6B. The highest univariate standard Z'-factor was 0.32, and the highest robust Z’-factor was 0.54, both for the parameter “% 3 or more peaks”, in concordance with the strong reduction of the number of oscillations upon IκBα knockdown. According to established criteria [29], a Z'-factor >0.5 indicates an assay suitable for HTS. The high values for the robust Z’-factors that were obtained therefore may validate our method for HTS. By calculating the linear projection for the parameters with Z'-factor >0, in this case 6 parameters, a satisfactory multivariate Z'-factor of 0.61 and a very good robust Z'-factor of 0.78 were obtained which further validates our assay for HTS (Figure 6C).

## Discussion and Conclusions

Controlling cellular fate in response to external stimuli is an important event in many physiological and pathological processes and in the action pharmacologically active compounds. Signaling routes that are involved herein frequently modulate gene-transcription by activation of nuclear transcription factors, such as NF-κB. In order to obtain a better insight in underlying processes that lead to the activation of these transcription factors. their subsequent translocation to the nucleus, and in the downstream events that follow their activation, methods need to be developed that enable the study of such events at the individual cell level and in high throughput fashion. In this study, we successfully developed such a methodology based on a novel method for cytoplasm definition (BEVC) and nuclei segmentation (WMC). Our method can easily be adapted to study the activation and nuclear cycling of other nuclear transcription factors as well.

The cell line used in this study (HepG2) is an epithelial-like hepatoma cell line, showing clustered and stacked cell growth. This influences the readout for GFP-p65 translocation by epifluorescence microscopy: superimposed, yet out of focus nuclei decrease the accuracy of single cell tracking and measurements. By adopting confocal microscopy in this study, the resolution and accuracy of single cell measurements are increased. Furthermore, we introduced the BEVC algorithm for accurate cytoplasm definition based on cell topology. Combined with WMC segmentation for the nuclear mask, and a series of quantification processes such as linear interpolation, the NF-κB translocation profile of each individual cell can be constructed. In order to validate our method, three sets of tests were applied on 5 time-lapse image series. These tests evaluated the proposed quantification method from three different perspectives, i.e. (1) accuracy of BEVC algorithm, (2) accuracy of calculated NF-κB translocation profiles, and (3) correct identification of cell sub-populations. In our test, only 10% of cells were segmented incorrectly by BEVC algorithm. Compared with a 14% error rate obtained by the dilation method and a 12% error rate by the Voronoi method, we can state that the BEVC algorithm provides sufficiently accurate cell segmentation. The BEVC algorithm is also highly efficient, which is a key consideration for HTS analysis. Other algorithms, such as contours derived from an active shape model [33], would possibly define a more precise cell edge, yet at the cost of analysis speed. Moreover, due to the uniform distribution of GFP-p65 in the cytoplasm, exact detection of cell boundaries is considered less relevant.

In the second and third validation test, we evaluated the accuracy of the calculated NF-κB translocation profiles and the accuracy of cell sub-population identification respectively. Our results indicate no significant differences between human generated benchmarks and the results obtained from the automated computational procedures, thereby validating our methods for studying NF-κB translocation, not only in the context of overall effects on the translocation response, but more importantly, also at the individual cell level.

In order to establish the sensitivity of our algorithm for perturbation of the biological system, an experiment was performed by pre-exposing the HepG2/GFP-p65 cells for 2 hours with an IKK-inhibitor, BMS-345541 before TNFα stimulation, which will prevent NF-κB nuclear translocation. The results show that already at the lowest concentration of the IKK-inhibitor that was used, perturbation of nuclear translocation of NF-κB was observed, thus validating our method for studying factors that affect this translocation. We also validated that our quantification method can successfully be applied in the context of high-throughput functional genomics screening. For this screening, the approach of gene silencing by transient transfection of short interfering RNAs (siRNAs) was applied. Based on calculation of multivariate Z'-factors, we demonstrated that our NF-κB quantification method can be used in HTS assays to identify genetic players that interfere with the nuclear translocation of NF-κB.

Here we demonstrated the effect of IκBα silencing by siNFKBIA treatment on NF-κB oscillation. Theoretically the expected effect of IκBα loss would be persistent nuclear presence of NF-κB, however, this is not observed. The 3-day siRNA treatment instead led to an increased expression of the GFP-p65 construct, which was strongly retained in the cytoplasm, even upon TNFα stimulation (Figure 6A). Western-blot analyses showed that the loss of IκBα had resulted in a basic upregulation of NF-κB target genes, including A20 and IκBα itself (data not shown), indicating that upon siNFKBIA treatment, the reporter cells had undergone multiple rounds of NF-κB translocation that most likely prevented further activation at the time of imaging.

NF-κB signaling is a complex process, and the balance of cytokine production and intracellular signaling transduction controls cellular fate in innate immunity and inflammation responses [14], [34]. It has been established by several groups that the individual cell response to cytokines may be very heterogeneous and is characterized by a full response of a few cells at low TNFα concentrations, and a similar response, but now from almost all cells, at high concentrations, thereby creating distinct sub-populations of cells [10], [14], [15]. We show that in the HepG2 cells used in this manuscript, in a non-stimulated population, 5% of the cells will oscillate spontaneously. Spontaneous nuclear translocation has also been reported in neuroblastoma cells, although at a slightly higher level (18%) [15]. It is thought that this cellular variation serves biological important goals such as stability in acute tissue responses that are made up from highly heterogeneous individual oscillatory cell responses [14]. Therefore, it is an important goal and a major challenge to quantify cell sub-populations within the NF-κB response pathway. Several methods have been described that partially meet this demand [10], [14], [15], [35]. However, none of these is suitable for HTS because they either lack fully automated image analysis and require human intervention at some point, or require special equipment that prohibits massive parallel screening. The development of a methodology suitable for HTS in the context of NF-κB signaling as presented in this study, whereby time courses of NF-κB translocation can be recorder in hundreds of individual cells over a period of many hours, presents a major breakthrough in this field. It now becomes possible to identify factors that govern NF-κB signaling at a genome wide scale. We are currently performing siRNA screening using this model to identify novel kinases and ubiquitinases that affect TNF-induced NK-κB nuclear shuttling.

Finally, the analogue parameters that we acquire of all the individual translocation profiles can be used as variables to model the sinusoidal oscillation of NF-κB translocation by systems biology approaches [14], [15], [35].

## Materials and Methods

### Cell Line and Cell Culture

HepG2 cells stably expressing N-terminally GFP-tagged p65 (GFP-p65) [36] were maintained in Dulbecco’s modified Eagle’s medium (DMEM) with high glucose, 10% (v/v) FBS and 25 u/mL penicillin/25 µg/mL streptomycin. HepG2/GFP-p65 cells were seeded on Greiner micro-clear 96well black plates (20,000 cells/well) and grown at 37°C, 5% CO_2_ for 2–3 days.

### Treatment of Cells

The human cytokine TNFα (R&D Systems) was used in all experiments at 10 ng/mL. The IKK inhibitor BMS-345541 was from Sigma-Aldrich. Transient knock-down of NFKBIA was achieved using siGENOME NFKBIA SMARTpool siRNA (50 nM; Dharmacon Thermo Fisher Scientific, Landsmeer, the Netherlands) and transfected into the HepG2 cells 3 days before imaging with INTERFERin (Polyplus transfection, Leusden, the Netherlands). Transfections with siGENOME SMARTpool CASP8 siRNA were used as negative controls in these experiments. Prior to imaging, nuclei were labelled with 100 ng/ml Hoechst 33342 in culture medium for 45 minutes. For confocal fluorescence microscopy, upon recording the first frame of the time-series, TNFα was added as 10 µL to each well containing 190 µL medium.

### Fluorescence Microscopy

The NK-κB nuclear translocation in the HepG2/GFP-p65 cells was imaged using a Nikon TiE2000 microscope equipped with a Perfect Focus System at 37°C with 5% CO2 delivery to the sample plate location. Both the Hoechst-nuclear channel (excitation 405 nm, emission: 450 nm) and the GFP-p65 channel (excitation 488 nm, emission 515 nm) were recorded with the laser excitation confocal system. Images were acquired with a 20x (NA 0.75) dry Plan Apochromat objective and the image acquisition was controlled by EZ-C1 software (Nikon). In each well, an image from the same position was acquired every 6 minutes for a period of 6 hours. The time-lapse series were exported in TIFF files as 16-bit digital images with 512×512 pixel frames.

### Image Analysis and Statistical Analysis

Image analysis was implemented using ImageJ (http://rsbweb.nih.gov/ij/). In-house plugins were written for quantification of both translocation profile and analogue parameters (see file S1). R (http://www.r-project.org/) was used to calculate the ANOVA test, t-test and multiparametric Z'-factor.

## Supporting Information

Figure S1 **Nuclear mask validation.** (A) accurately identified nuclear masks overlapping with nuclei and (B) incorrect masks manually identified.
(TIF)

Figure S2 **10-fold cross validation result for 7 different classification methods.** The best result was obtained from quadratic Bayes normal classification when 2 features were selected (marked by red box).
(TIF)

Figure S3 **Cellular mask validation.** (A) Out focus region are formed when cells are clustered on top of each others (in the red box). (B) No nuclear masks were identified in those out of focus regions. (C) Very big Voronoi cells were generated due to the missing nuclear masks, and consequently big ellipses were generated (D). Overlap of the GFP channel with ellipses (D) clearly showed that those big ellipses contained multiple cells. An area threshold was trained to discard incorrect cellular masks.
(TIF)

Figure S4 **Validation of the automated NF-κB translocation quantification method.** (A–E). The cell numbers (#), standard deviation and mean of the time profile of the intensity ratio obtained from different individuals and computational result. (F) The accuracy validation results from the split-plot ANOVA analysis. Df: degrees of freedom. Sum Sq: sum of squares. Mean Sq: mean of squares.
(TIF)

Figure S5 **The outline to quantify the analogue parameter from individual time course profiles**. (A) One example time course profile of one cell. (B) Smoothed profile on which we defined the local maximum (peak of translocation) and local minimum (valley of translocation). (C) Afterwards, parameters were measured to characterize the profile dynamic.
(TIF)

File S1 **Validation of nuclear masks, cellular masks, and NF-κB translocation quantification method, and quantification of analogue parameters.**
(DOC)

Table S1 **Morphological parameters for training the nuclear classifier.**
(DOC)

Table S2 **Definition of analogue parameters measured for each individual cell translocation profile.**
(DOC)
