# Supplementary figures and images for: Automated Analysis of NF-κB Nuclear Translocation Kinetics in High-Throughput Screening
Source: PLoS One. 2012 Dec 27;7(12):e52337. doi: 10.1371/journal.pone.0052337 (PMC3531459; doi:10.1371/journal.pone.0052337)

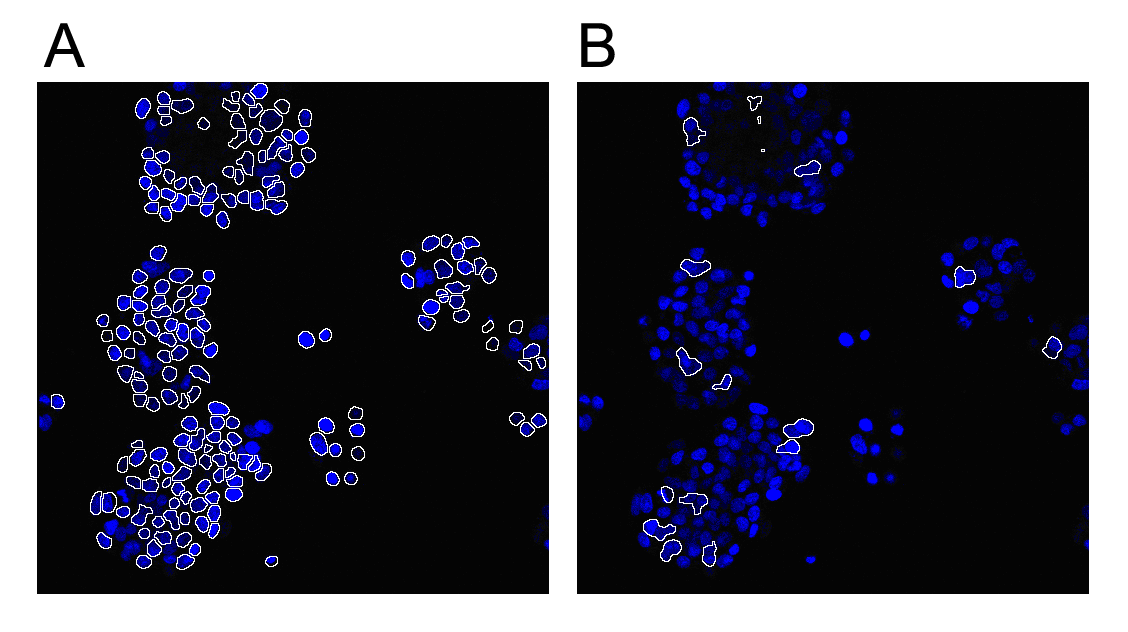

Supplement: Figure S1 — Nuclear mask validation. (A) accurately identified nuclear masks overlapping with nuclei and (B) incorrect masks manually identified. (TIF) [file pone.0052337.s001.tif]

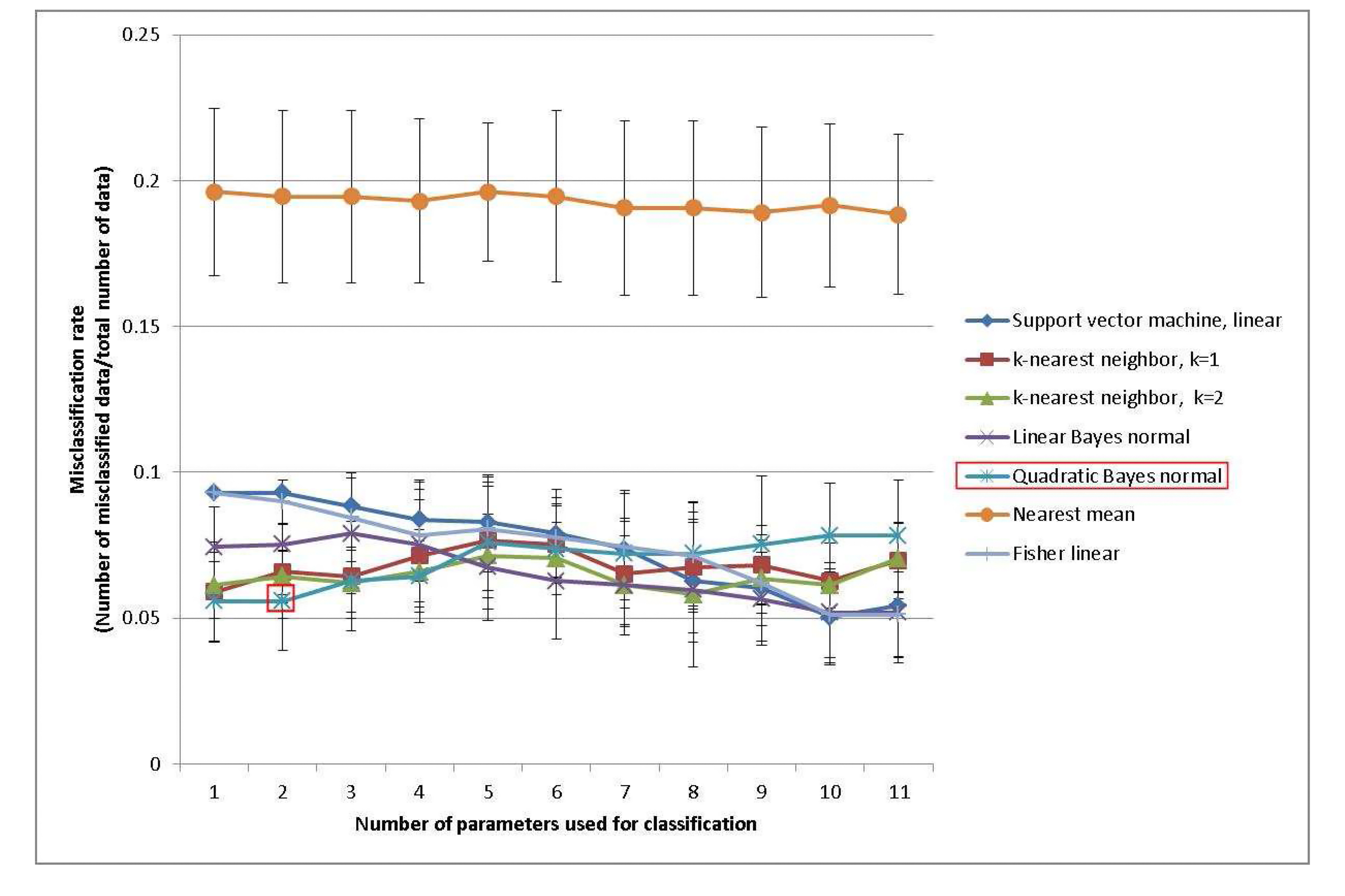

Supplement: Figure S2 — 10-fold cross validation result for 7 different classification methods. The best result was obtained from quadratic Bayes normal classification when 2 features were selected (marked by red box). (TIF) [file pone.0052337.s002.tif]

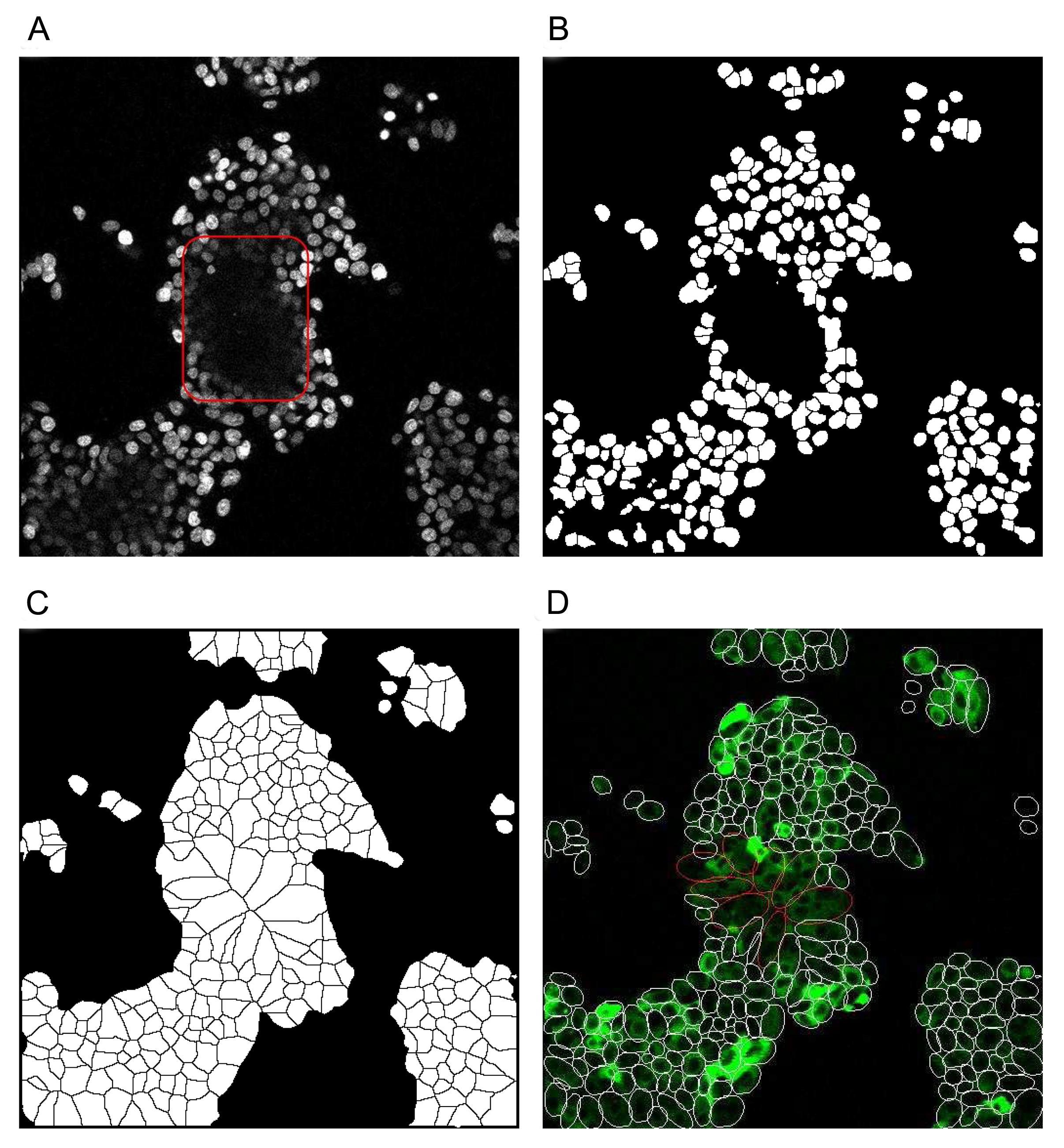

Supplement: Figure S3 — Cellular mask validation. (A) Out focus region are formed when cells are clustered on top of each others (in the red box). (B) No nuclear masks were identified in those out of focus regions. (C) Very big Voronoi cells were generated due to the missing nuclear masks, and consequently big ellipses were generated (D). Overlap of the GFP channel with ellipses (D) clearly showed that those big ellipses contained multiple cells. An area threshold was trained to discard incorrect cellular masks. (TIF) [file pone.0052337.s003.tif]

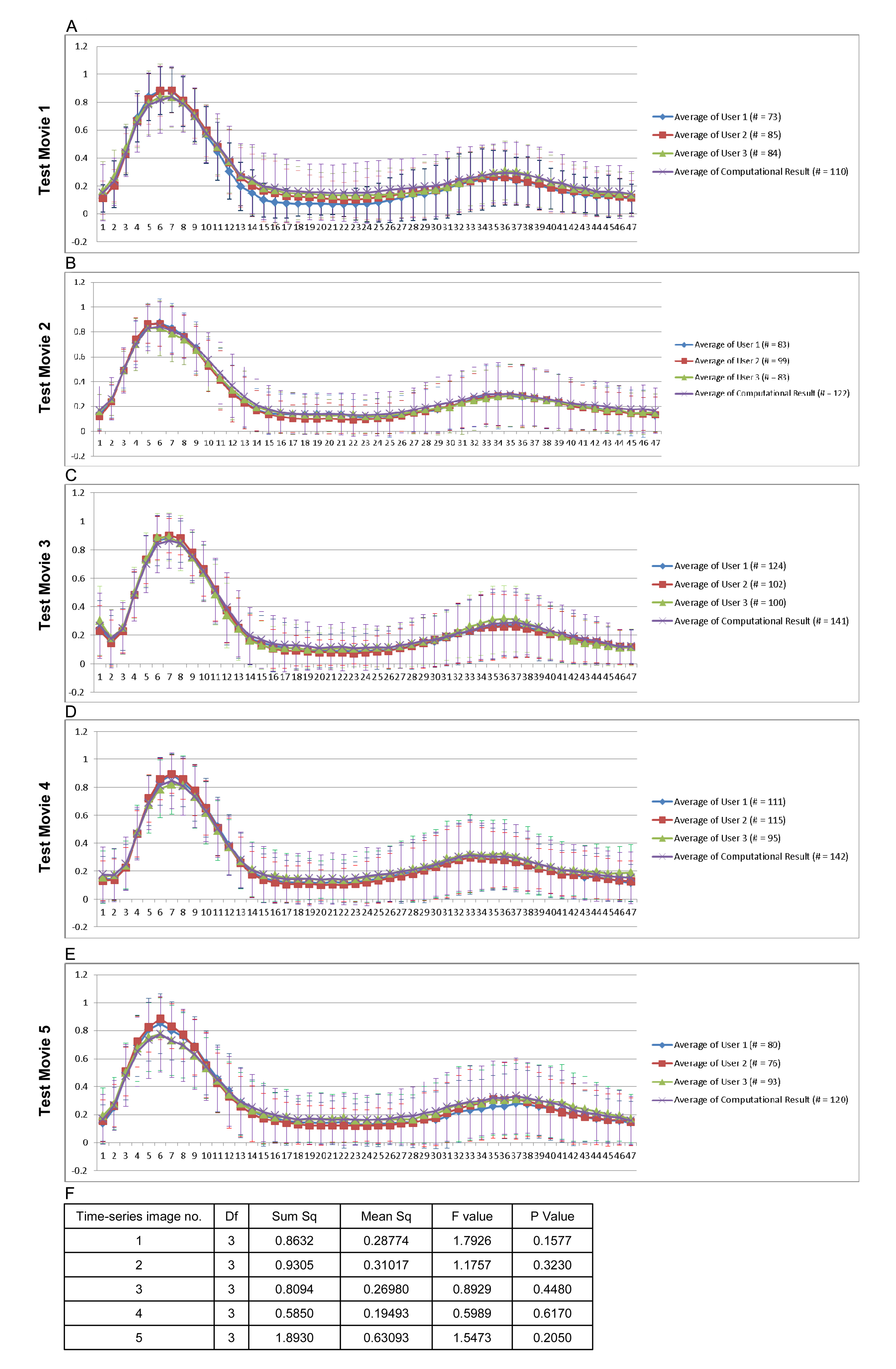

Supplement: Figure S4 — Validation of the automated NF-κB translocation quantification method. (A–E). The cell numbers (#), standard deviation and mean of the time profile of the intensity ratio obtained from different individuals and computational result. (F) The accuracy validation results from the split-plot ANOVA analysis. Df: degrees of freedom. Sum Sq: sum of squares. Mean Sq: mean of squares. (TIF) [file pone.0052337.s004.tif]

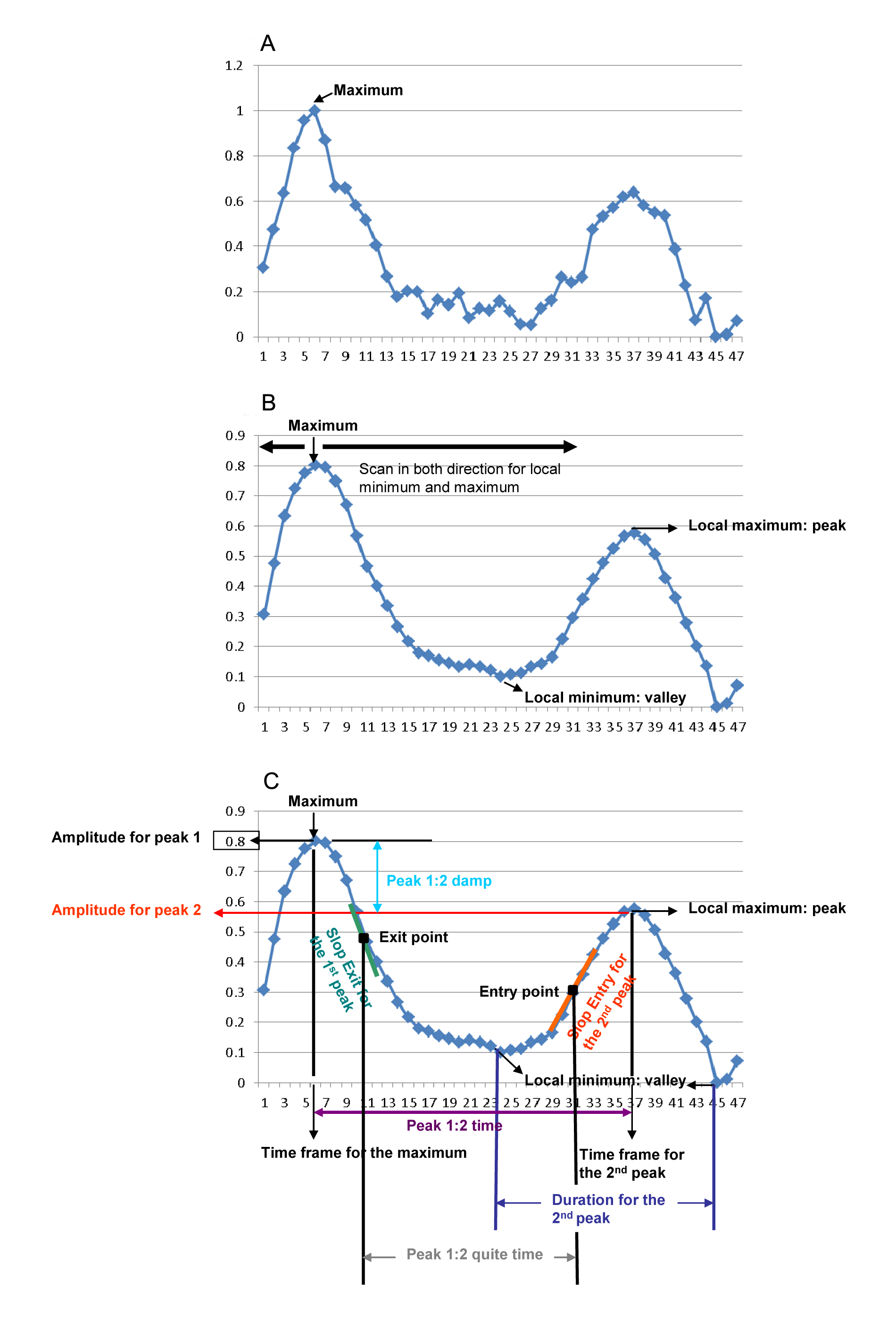

Supplement: Figure S5 — The outline to quantify the analogue parameter from individual time course profiles. (A) One example time course profile of one cell. (B) Smoothed profile on which we defined the local maximum (peak of translocation) and local minimum (valley of translocation). (C) Afterwards, parameters were measured to characterize the profile dynamic. (TIF) [file pone.0052337.s005.tif]
