# Supplementary material for: Automated Analysis of NF-κB Nuclear Translocation Kinetics in High-Throughput Screening
Source: PLoS One. 2012 Dec 27;7(12):e52337. doi: 10.1371/journal.pone.0052337 (PMC3531459; doi:10.1371/journal.pone.0052337)
Supplement: File S1 — Validation of nuclear masks, cellular masks, and NF-κB translocation quantification method, and quantification of analogue parameters. (DOC) [file pone.0052337.s006.doc]

## 1. Nuclear mask validation by classification

### a. Overview

After defining the nuclear mask by watershed masked clustering (WMC), we observed that some masks were not segmented accurately. Especially masks that extent over nuclear boundaries would lead to the inaccurate average nuclear intensities. To solve this problem, we decided to train a classifier which can automatically recognize enlarged nuclear masks and discard them. This classifier can be used for experiments carried out on different dates or with different treatments, as long as the microscope settings and cell line remain the same.

### b. Training data

5 frames were randomly selected from 5 different time lapse movies. Then incorrect nuclear masks were selected manually. For all test images, 1179 nuclear masks were validated as accurate segmentation result (e.g. Supporting Figure S1A), and 127 nuclear masks were considered as incorrect (e.g. Supporting Figure S1B). Next morphological parameters (Table S1) were calculated using ImageJ, on both correct masks and incorrect masks, then used to train the classifier.

### c. Classification

Feature selection was performed to avoid the curse of dimensionality, using search algorithm ‘forward’. To define the optimal number of features, multiple classification methods were applied and a 10 fold cross-validation was used to evaluate the number of features, as well as the accuracy of each classification algorithm. The classification methods we tested were k-nearest neighbor classification with k = 1 or 2, linear Bayes normal classification [1,2,3], quadratic Bayes normal classification [1,3], nearest mean classification, fisher linear classification [1,3,4] and support vector machine with linear kernel. Supporting Figure S2 showed the cross-validation error rate for each classification method calculated on certain number of features. The result showed that when 2 features were selected, quadratic Bayes normal classification gave quite low error rate 5.58%. Those 2 features are circularity and area. In the end, a quadratic classifier was obtained which can be used to automatically validate the nuclear mask for the whole experiment. All the functions were implemented using PRtools on MATLAB.

## 2. Cellular mask validation by classification

### a. Overview

We also have validated the cellular masks. Since we used the best-fit ellipse of Voronoi cell to simulate the cellular region, the circularity for each mask would all be 1, so that can not be used for the classification. Therefore we decided to only use an area threshold to identify the incorrect cellular masks. For regions where cells grow on top of each other, the thickness of the sample is bigger than the depth of field of the microscope and therefore the images are not in focus in those regions (Supporting Figure S3A). As a result, nuclei can not be detected and no nuclear masks were obtained in those regions (Supporting Figure S3B). Consequently, very big Voronoi cells (Supporting Figure S3C) and ellipses are generated (Supporting Figure S3D). Overlap of the GFP channel with ellipses clearly showed that those big ellipses contained multiple cells. To discard those incorrect cellular masks, users first manually distinguished those ellipses (marked in red) from the rest (marked in white) to get the training data. Next, an area threshold was trained to validate the cellular masks.

## b. Training data and classification

5 images which contain out of focus regions were chosen. Next, incorrect cellular masks which covered multiple cellular areas where the corresponding nuclear masks were missing were manually identified. Afterwards, the areas of these identified masks were measured, as well as those of correctly identified masks. In the end, an optimal area threshold was set up to minimize false classification.

## 3 Validation of the automated NF-κB translocation quantification method

We validated our NF-κB translocation quantification method by comparing automatically generated translocation profiles with a benchmark which was produced from cells with confirmed correct segmentation and tracking by human perception. 5 randomly selected time lapse movies were used and each of them had 47 frames. From each test movies, 3 benchmarks were generated separately by 3 independent individuals (Figure S4A-S4E), in order to compensate for possible human bias. Subsequently, a split-plot ANOVA was performed to test for the difference between the benchmark profiles generated by the 3 test persons and the computational result, in total 4 groups. The metric is the NF-κB Nuclear/Cytoplasmic intensity ratio, and 2 independent factors are time and group. There are no significant differences between the benchmarks and the computational result (Figure S4F). This indicates that the designed algorithm provides an accurate estimation of NF-κB translocation profiles.

## 4 Quantification of analogue parameters

### a. Overview

One advance of our fully automated method is its ability to provide analogue parameters automatically for each time course profile. These analogue parameters translate the profiles into numerical parameters, such as number of peaks and amplitude of each peak (Supporting table S2). This is very useful for categorizing different cell subpopulations according to their analogue parameters so that we can study not only the influence of various conditions on the whole population but also on subpopulations.

The outline for the quantification is shown in the Figure S5. For each time course profile, we first located the maximum value. Then each profile was smoothened to remove the small spikes which may be caused by intensity noise. Next starting from the maximum point, we scan in both directions along the translocation profile, to search for the neighboring local minimum and local maximum. After defining all local maxima and local minima which represent peaks and valleys of each nuclear translocation events, parameters were measured to characterize each translocation event.

## b. Smoothening of each time course profile

The main idea of smoothing is to remove noisy spikes on each profile so that we can locate the local maximum and local minimum precisely. We used a 1xN mean filter window (see below) to slide, frame by frame, convolving over the entire profile. N can be determined experimentally.

A. B.

1

1

1

1

1

-1

0

1

An example filter with window size 1x5 (A); nuclei entry and exit frame detection (B).

## c. Local maximum and local minimum

Logically, local maximum and local minimum should appear alternatively. Combining this rule with following 4 rules, we experimentally define a point local minimum.

1) The N:C ratio of this point is smaller than neighbor points;

2) The adjacent local maximum should be at least 2 frames away from this point;

3) The N:C ratio of this point should be below 0.5;

4) The ratio difference between adjacent local maximum and this point should be at least 0.1

For the local maximum, the rules are:

1) The N:C ratio of this point is bigger than neighbor points;

2) The adjacent local minimum should be at least 2 frames away from this point;

3) The ratio difference between adjacent local minimum and this point should be at least 0.1

According to above rules, we are giving pseudo code as following:


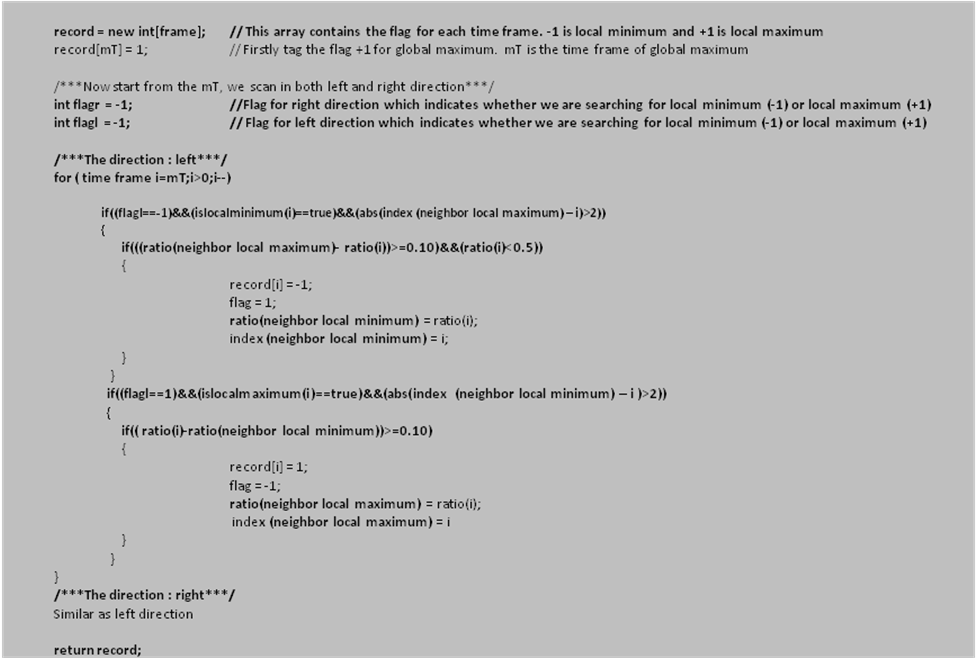


## d. Nuclei entry and exit time points

Some of parameters, such as SlopEntry and SlopExit of each translocation event, require us first to define where nuclei entry and exit time points are (Supporting Figure S5C), then calculate the gradient on that point. Those parameters can provide the information about how fast NF-κB translocates into or exits nuclei. The method to calculate them is similar to the 2D edge detection by Sobel operator. One filter (Supporting Figure S5B) is convolved over the smoothed profile to calculate the gradient approximation for each frame, and then the frames with local maximum magnitude were assigned as SlopeExit or SlopeEntry according to the direction of the gradient.

# References

1. Liu CJ, Wechsler H (2000) Robust coding schemes for indexing and retrieval from large face databases. IEEE Transactions on Image Processing 9: 132-137.

2. Duda RO, Hart PE, Stork DG (2001) Pattern Classification. 2nd edition. New York: John Wiley and Sons Inc. 91 p.

3. Raudys S, Duin RPW (1998) Expected classification error of the Fisher linear classifier with pseudo-inverse covariance matrix. Pattern Recognition Letters 19: 385-392.

4. Webb AR (2002) Statistical pattern recognition. 2nd edition. West Sussex (United Kingdom): John Wiley and Sons Inc. 496 p.
