# Supplementary material for: Automated Analysis of NF-κB Nuclear Translocation Kinetics in High-Throughput Screening
Source: PLoS One. 2012 Dec 27;7(12):e52337. doi: 10.1371/journal.pone.0052337 (PMC3531459; doi:10.1371/journal.pone.0052337)
Supplement: Table S1 — Morphological parameters for training the nuclear classifier. (DOC) [file pone.0052337.s007.doc]

| Index | Measurement | Description |
| --- | --- | --- |
| 1: | Area | Number of pixel in the current mask |
| 2: | Perimeter | The length of the outside boundary of the current mask |
| 3: | MajorAxis | The primary axis of the best fitting ellipse of the current mask |
| 4: | MinorAxis | The secondary axis of the best fitting ellipse of the current mask |
| 5: | Angle | The angle between the primary axis and a line parallel to the X-axis of the image |
| 6: | Circularity | $4\pi\times\frac{[Area]}{[Perimeter]^{2}}$ |
| 7: | Maximum caliper | The longest distance between any two points along the boundary of the current mask |
| 8: | Minimum caliper | The shortest distance between any two points along the boundary of the current mask |
| 9: | Axis ratio | $\frac{[MajorAxis]}{[MinorAxis]}$ |
| 10: | Roundness | $4\times\frac{[Area]}{\pi\times[MajorAxis]^{2}}$ |
| 11: | Solidity | $\frac{[Area]}{[ConvexArea]}$,  where ConvexArea is the pixel number of the minimum convex hull containing the current mask |
