# Supplementary material for: Automated Analysis of NF-κB Nuclear Translocation Kinetics in High-Throughput Screening
Source: PLoS One. 2012 Dec 27;7(12):e52337. doi: 10.1371/journal.pone.0052337 (PMC3531459; doi:10.1371/journal.pone.0052337)
Supplement: Table S2 — Definition of analogue parameters measured for each individual cell translocation profile. (DOC) [file pone.0052337.s008.doc]

| **Analogue parameters** | **Parameter description** |
| --- | --- |
| Number of peaks | Number of of nuclear translocation events shown in the translocation profile |
| Time for max | The time when N:C localization reaches the maximum along the profile |
| Time(peak 1) | The time when N:C localization reaches the first translocation maximum of the profile |
| Amplitude (peak 1) | The N:C localization ratio of the first translocation maximum |
| Duration(peak 1) | Duration of first nuclear translocation event |
| SlopeEntry(peak 1) | Slope of nuclear entry of the first nuclear translocation event |
| SlopeExit(peak 1) | Slope of nuclear exit of the first nuclear translocation event |
| Time(peak 2) | The time when N:C localization reaches the second translocation maximum of the profile |
| Amplitude(peak 2) | The N:C localization ratio of the second translocation maximum |
| Duration(peak 2) | Duration of second nuclear translocation event |
| SlopeEntry(peak 2) | Slope of nuclear entry of the second nuclear translocation event |
| SlopeExit(peak 2) | Slope of nuclear exit of the second nuclear translocation event |
| Time(peak 3) | The time when N:C localization reaches the third translocation maximum of the profile |
| Amplitude(peak 3) | The N:C localization ratio of the third translocation maximum |
| Duration(peak 3) | Duration of third nuclear translocation event |
| SlopeEntry(peak 3) | Slope of nuclear entry of the third nuclear translocation event |
| SlopeExit(peak 3) | Slope of nuclear exit of the third nuclear translocation event |
| Peak 1:2 time | The duration time between the first translocation maximum and the second maximum |
| Peak 1:2 damp | The difference of N:C ratio between the first translocation maximum and the second maximum |
| Peak 1:2 quiet time | Time between end of the first nuclear exit and start of next nuclear entry event |
| Peak 2:3 time | The duration time between the second translocation maximum and the third translocation maximum |
| Peak 2:3 damp | The difference of N:C ratio between the second translocation maximum and the third translocation maximum |
| Peak 2:3 quiet time | Time between end of the second nuclear exit and start of next nuclear entry event |
| Average amplitude | Average N:C ratio of all the translocation maxima |
| Average minima | Average N:C ratio of all the translocation minima |
| Nuclear occupancy time | Total amount of time when the N:C localization ratio> 0.5 |
